# A Tunable Graphene Superlattice with Deformable Periodical Nano-Gating

**DOI:** 10.3390/nano14121019

**Published:** 2024-06-13

**Authors:** Binbin Wei, Haosong Ying, Junrong Chen, Qing Zang, Jiduo Dong, Hao Zhang, Yang Liu, Chunheng Liu

**Affiliations:** 1 Institute of System Engineering, Beijing 100091, China; zangqing348@163.com (Q.Z.); liuchunheng@126.com (C.L.); 2 Department of Physics, Harbin Institute of Technology, Harbin 150001, China19b911015@stu.hit.edu.cn (J.D.); 19b911016@stu.hit.edu.cn (H.Z.)

**Keywords:** tunable graphene superlattice, nano-gating, band engineering

## Abstract

Graphene superlattices have simple and controllable electronic band structures, which can also be electrostatically tuned. They have been widely studied for band engineering and strong correlated physics, and have led to the discovery of a variety of exciting phenomena. To experimentally study the physics of graphene superlattices in a systematic way, it is desirable to control the structure parameters, which barely exist at the moment, onsite. Here, a tunable superlattice with graphene and a deformable gating structure is demonstrated. The period and duty cycle of the nano-gating, and furthermore of the superlattice potential, can be tuned through altering the shape of the gating structure with piezo-actuators, offering a tunable band structure. The tuning of the electronic band structures of both a two-dimensional and a one-dimensional superlattice is demonstrated with numerical simulations, offering a new approach for tunable electronic and photonic devices.

## 1. Introduction

Superlattices (SLs) can be used as an artificial crystal for electrons. Unlike in natural crystals, where the period and the strength of the potential field are fixed, the parameters of the potential field in an SL can be designed and manipulated. Therefore, SLs offer an effective approach to constructing and controlling the energy band structure of electrons. Recently, it has been found that graphene is an ideal material for creating synthetic band structures using SL patterning. As a 2-dimensional (2D) material, graphene possesses surface electrons, which can be easily manipulated with an external potential field. Furthermore, due to its linear band dispersion at low energy, graphene SLs (GSL) show peculiar properties, especially the cyclic flattening/unflattening of the Dirac cone as the strength of the SL potential field increases, along with the flat energy band induced by it [1,2,3]. These are highly tunable flat bands in which the group velocity of electrons is zero and the density of states is large, resulting in strong electron correlations [4]. Strong correlations make it possible to achieve many many-body ground states. Up to now, unconventional superconductivity [5], correlated isolator behavior [6], unconventional ferroelectricity [7], ferromagnetism [8,9] and the quantum anomalous Hall effect [8,9,10] have been demonstrated with GSLs. Although GSLs have been widely studied in recent years, most of the reported GSLs have fixed structure parameters, and in situ structure parameter tuning methods are lacking for GSLs. This poses difficulties in both experimental and theoretical studies of GSLs and limits their potential for application. Experimentally, the restricted available parameter space cannot provide a broad view of the relationship between the band structure and strong correlations; theoretically, the lack of systematic measurement data makes it impossible to identify the essential degrees of freedom to capture the physics [4].

The reported structure parameter tunable GSLs are all based on twisted GSLs. An atomic force microscope (AFM) or a polymer gel handle can be used to tune the twistable angle of bilayer graphene or the angle between the h-BN layer and monolayer graphene [11,12,13]. In addition, the effective twist angle of bilayer graphene can be modulated using the longitudinal electric fields available in a waveguide [14]. For all these approaches, both the achievable structure parameter range and the unit cell shape are determined and limited by twisted GSLs.

In this paper, a tunable GSL based on deformable periodical nano-gating is proposed. It is inspired by the deformability of conformal graphene supported with polydimethylsiloxane (PDMS) [15,16]. The period of the nano-gating and the contact area between the gating unit cell and the dielectric layer can be adjusted by applying a force on the gating with piezoceramics (PZT). Therefore, both the period and the duty cycle of the GSL can be tuned onsite.

## 2. Materials and Methods

**Figure 1:**
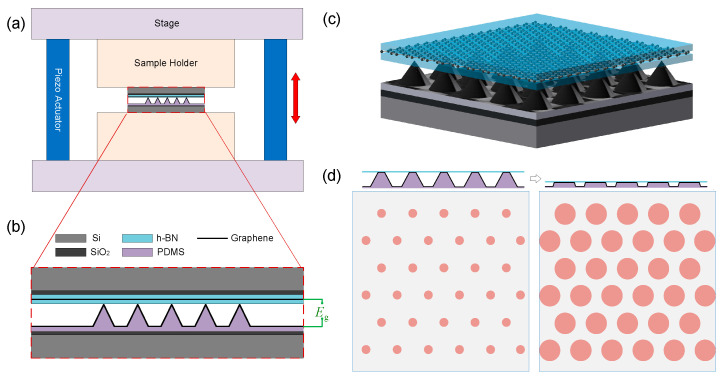
Schematic of the tunable GSL device and its working principle. (**a**) Structure of a complete device. It is composed of two parts: the sample platform (stage, sample holder, piezo actuator) and GSL. The distance between the two stages along the red arrow can be adjusted by applying a voltage on the piezo actuator. (**b**) Schematic of the GSL. (**c**) Three-dimensional illustration of the GSL. (**d**) Variation in the carrier density distribution at different PZT voltages.

A complete tunable GSL device includes a sample platform and the GSL, as shown in Figure 1a. The GSL is composed of the deformable periodical nano-gating and a h-BN/graphene/h-BN sandwich structure, as shown in Figure 1b. To show the structure of the GSL more clearly, a 3-dimensional illustration of it is given in Figure 1c, where the pyramids are the deformable periodical nano-gating, which is patterned in a triangle array. Therefore, a triangle superlattice with circle unit cells on the channel graphene is defined. When varying the PZT voltage, the distance between the h-BN layer and the PDMS layer changes, which in turn modifies the deformation of the nano-gating and the current density distribution. Figure 1d gives a schematic of the carrier density map for the channel graphene at two different gating deformations while the gate voltage is fixed. The carrier density is zero in the grey area, while tunable by the gate voltage in the red area. As can be seen, the duty cycle (the ratio between the diameter of the contacting area and the superlattice period) is increased when the nano-gating is compressed. Unlike twist GSLs, the shape and size of the gating unit cell can be designed for the proposed device. Furthermore, devices with different configurations can be fabricated as an array and measured together in one cooling cycle, which would improve the efficiency of experiments significantly.

## 3. Results

### 3.1. Superlattice Potential Distribution

For the tunable superlattice, the direct effect of the nano-gating is the creation of a periodic, fluctuating carrier density n(x,y). This leads to an SL potential of

(1) $$U_{\mathrm{SL}}(x,y)=-\mathrm{sgn}[n(x,y)]\hbar v_{F}\sqrt{\pi \left|n(x,y)\right|}$$

**Figure 2:**
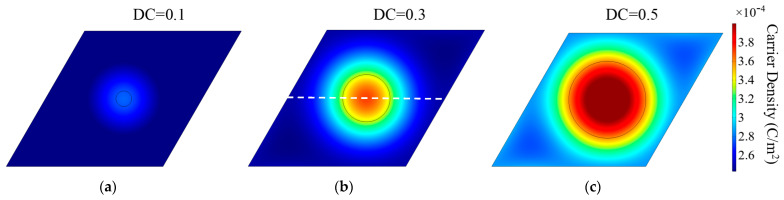
Carrier density distribution for a triangle GSL with a 40 nm period at different duty cycles, namely 0.1, 0.3, 0.5, for (**a**), (**b**), (**c**), respectively. The hBN thickness is 10 nm. The linecut in Figure 3 is done across the white dashed line in (**b**).

Here, a positive carrier density means that the carriers are electrons, while a negative carrier density means that the carriers are holes. To analyze the tunability of the proposed GSL device, its gating-induced surface carrier density distribution is simulated with the COMSOL Multiphysics 6.1 AC/DC module and used to obtain the SL potential. Here, due to the periodicity, only one unit cell of the GSL is considered and the details of the simulation model are given in the Supplementary Information. A triangle GSL with a period of 40 nm is discussed in this paper. The influence of the GSL duty cycle and hBN thickness is analyzed. The duty cycle affects the diameter of the circular contact areas, being 10 nm, 12 nm and 20 nm for a duty cycle of 0.1, 0.3 and 0.5, respectively. Figure 2 shows the carrier density distribution of the GSL at different duty cycles, with the gate voltage being 0 V and the balance potential (V_b_ in Figure S1) being 0.5 V. When increasing the duty cycle, the amplitude of the carrier density and the covering area of the carriers increase as well.

**Figure 3:**
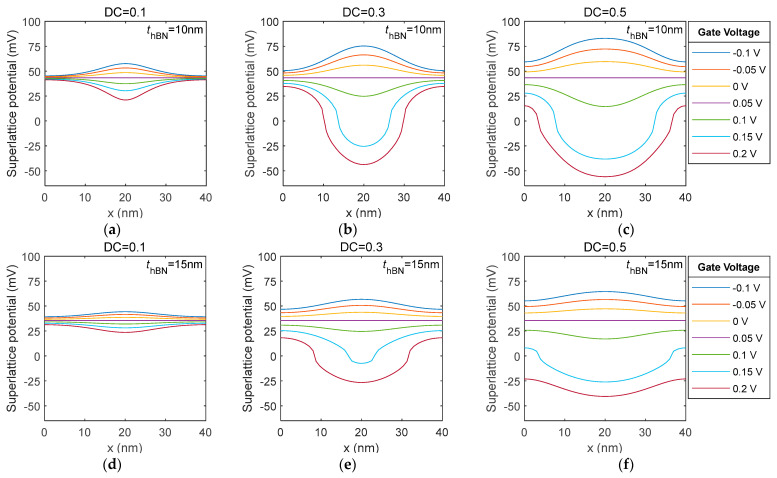
Line-cut of the superlattice potential distribution for a triangle GSL with a 40 nm period at different gate voltages and hBN thicknesses when the balance potential is 0.05. hBN thickness is 10 nm: (**a**–**c**). hBN thickness is 15 nm: (**d**–**f**). Lines with different colours are the results for different gate voltages.

The SL potential can then be obtained with Equation (1). To reveal the SL potential distribution straightforwardly, a line-cut is performed along the white dotted line in Figure 2b. The results show that the thickness of the hBN layer, the duty cycle and the gate voltage all affect the potential distribution of the superlattice, as shown in Figure 3. When the duty cycle is 0.1, the potential distribution is unipolar for both hBN thicknesses. At higher duty cycles (0.3, 0.5), the potential distribution is bipolar when the gate voltage is around three times the balance potential (0.15 V). Therefore, for the tunable superlattice, the potential distribution of the superlattice can be actively designed by setting the duty cycle and the gate voltage.

### 3.2. Mini-Band Structure at Different Duty Cycles

The mini-band structure of the GSL is obtained with the continuum model. The Hamiltonian for a 2-dimensional triangle GSL near the K/K′ valley can be written as

(2) $$H=H_{0}+V(r)$$

where

(3) $$H_{0}=\hbar v(\sigma_{x}k_{x}+\xi \sigma_{y}k_{y})=\hbar v\left[\begin{array}{cc} 0 & k_{x}-i\xi k_{y}\\ k_{x}+i\xi k_{y} & 0 \end{array}\right]$$

Here, $\sigma_{x}$ and $\sigma_{y}$ are the Pauli matrices ξ=±1 for K and K′ valleys, and

(4) $$V(r)=V_{s}\sigma_{0}\sum_{i=1}^{3}2\cos(G_{i}\cdot r),G_{i}=\frac{4\pi }{3a}\left(\cos\frac{i\pi }{3},\;\sin\frac{i\pi }{3}\right)$$

where $G_{i}$ is the reciprocal vector for a triangular superlattice. The Hamiltonian can be written in the momentum space with the basis $k_{\mathrm{mn}}$ and $k_{\mathrm{pq}}$. $k_{mn}=k+mG_{0}+nG_{1}$ and $k_{pq}=k+pG_{0}+qG_{1}$.m, n, p, q∈ℤ and k are the wave vectors within the first Brillouin zone. The Hamiltonian then becomes

(5) $$H_{mn,pq}(k)=\sum_{r}H_{0}(k_{mn})e^{i(k_{mn}-k_{pq})r}+V_{s}\sum_{i=1}^{3}\sum_{r}2\cos(G_{i}\cdot r)\sigma_{0}e^{i(k_{mn}-k_{pq})r}$$

**Figure 4:**
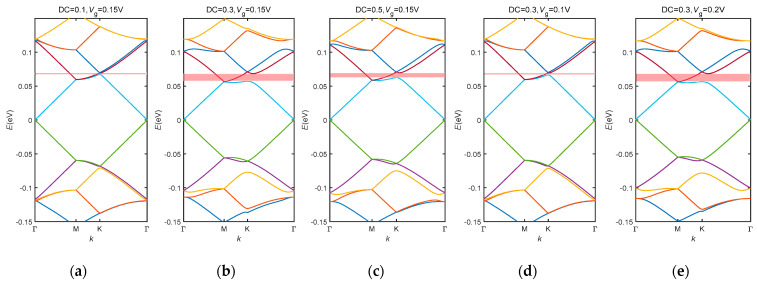
Band structure of a triangle tunable GSL at different gate voltages and duty cycles: (**a**) DC = 0.1, V_g_ = 0.15 V; (**b**) DC = 0.3, V_g_ = 0.15 V; (**c**) DC = 0.5, V_g_ = 0.15 V; (**d**) DC = 0.3, V_g_ = 0.1 V; (**e**) DC = 0.3, V_g_ = 0.2 V. DC stands for duty cycle, V_g_ is the gate voltage. The red shadowed areas in the figures show where the band gap is opened up. Lines with different colours correspond to different eigen values of the SL Hamiltonian.

For low-energy situations, it is possible to achieve the convergence when truncating the infinite large matrix $H_{mn,pq}(k)$ with m, n, p, q∈[−4,4] [17]. The Eigen energies of the GSL can then be obtained by diagonalizing $H_{mn,pq}(k)$. The GSL discussed here is the same one as mentioned in Section 3.1. The band structures of the GSL at different gate voltages and duty cycles are displayed in Figure 3. It is interesting to see that a bandgap is present at the secondary Dirac Point around 60 meV at K point. At a fixed gate voltage, the band gap first opens up and then drops down when the duty cycle is increased (Figure 4a–c, highlighted by red in the figures). At a fixed duty cycle, this band gap is broadened when the gate voltage is increased (Figure 4b,d,e).

**Figure 5:**
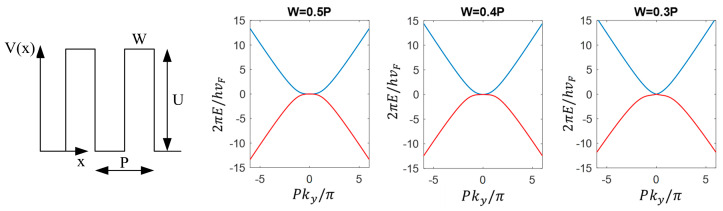
Band structure of a one-dimensional graphene superlattice in the *k*_y_ direction for *k*_x_ = 0 at different potential duty cycles when the potential amplitude is *u* = *UP*/*V*_F_ℏ = 4π. The blue curve and the red curve are the conduction band and the valence band, respectively.

For a 1-dimensional tunable GSL, where the electric potential varies periodically along one axis only, as expected, the duty cycle (the ratio between the barrier width W and period P of the SL potential, Figure 5) affects the symmetry of the band structures. As shown in Figure 5, the band structures of a 1-dimensional tunable GSL at three different duty cycles are calculated with the method reported in reference [18]. It is interesting to see that a flat band appears at the K point when the superlattice potential is *u* = *UP*/*V*_F_ℏ = 4π, where *U* is the barrier height, *V*_F_ is the fermi velocity and ℏ is the reduced Planck constant. At the duty cycle of 0.5, the conduction band and the valence band are symmetric. As the duty cycle decreases, the two bands become asymmetric.

### 3.3. Mini-Band Structure at Different Superlattice Periods

**Figure 6:**
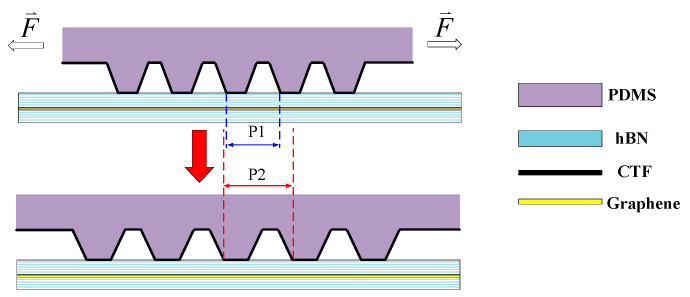
When the nano-gating is stretched or compressed in the horizontal direction with a force, the GSL period changes from P1 to P2 or vice versa, where CTF stands for the conducting thin film.

**Figure 7:**
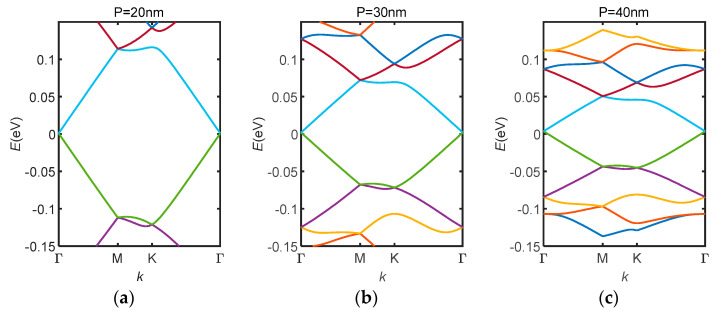
Electronic band structure for GSLs with different periods (P is the superlattice period): (**a**) P = 20 nm; (**b**) P = 30 nm; (**c**) P = 40 nm. Lines with different colours correspond to different eigen values of the SL Hamiltonian.

Apart from tuning the duty cycle of the potential field, its period can also be tuned by stretching or compressing the gating structure in the horizontal direction, as shown in Figure 6. This is especially effective for 1-dimensional superlattices. Varying the period of the superlattice potential offers an extra degree of freedom when tuning both the electronic and photonic properties of the GSL. For electrons, this compresses the electronic band structure (Figure 7a–c), thus lowering the band gap and changing the conducting property of the superlattice. For photons, this shifts the working frequency of an optoelectronic device when considering it as a plasmonic photonic crystal.

## 4. Conclusions

In conclusion, a tunable GSL is demonstrated in this work. Its period and duty cycle can be tuned with a nano-gating structure. Together with the gate voltage, they affect the amplitude and space distribution of the superlattice potential, thus changing the electronic and photonic band structures of the superlattice. It is demonstrated that the superlattice potential of a tunable triangle GSL turns from unipolar into bipolar when the duty cycle increases from 0.1 to 0.3 and 0.5 at a gate voltage of around three times the balance potential. The bipolar superlattice potential distribution means that the carrier type in the SL evolves along the space. Therefore, there will be a boundary between the electron area and the hole area. For electrons, this means a stronger modulation and richer physics. For photons, this might induce surface plasmon polaritons. As the period of SL can be tuned, the plasmon wavelength can be adjusted as well. Regarding the electron band structure, a band gap is opened up at the secondary Dirac point at around 60 meV at the K point. The gap width can be tuned with both the duty cycle and the gate voltage. In addition, as expected, the period of the superlattice can also be tuned by stretching or compressing the nano-gating in the horizontal direction, which in turn compresses or stretches the electronic band structure. For a 1-dimensional superlattice, the duty cycle alters the symmetry of the band structure.

In applied physics, tunable GSLs can serve as topological insulators for electrons and photons, giving rise to a variety of novel optoelectronic devices. As for fundamental physics, tuning the structure parameters, the gating voltage and magnetic field jointly builds a 3-dimensional parameter space for GSL. This facilitates a systematic study of the relationship between the band structure and transport properties, creating opportunities for discovering new physics. To date, it is still challenging to fabricate PDMS nanostructures with a period as low as 40 nm. The best reported result is 78 nm [19]. This is promising as, for photonic applications, 78 nm is already sufficient for photonic band engineering. For electrons, if the uniformity of the nanostructures is high, the disorder is low, and the mini-Brillouin zone edge can still be observed; this means that the electronic band structure can be tuned.

## Data Availability

The data presented in this study are available on request from the corresponding author.

## Supplementary Materials

The following supporting information can be downloaded at: https://www.mdpi.com/article/10.3390/nano14121019/s1, Text S1: Modelling in COMSOL; Text S2: Electrostatics Theory; Text S3: Material properties; Figure S1: Schematic diagram of a unit cell of the triangular superlattice in COMSOL.
