# Supplementary material for: A Tunable Graphene Superlattice with Deformable Periodical Nano-Gating
Source: Nanomaterials (Basel). 2024 Jun 13;14(12):1019. doi: 10.3390/nano14121019 (PMC11206335; doi:10.3390/nano14121019)
Supplement: Supplementary file 1 [file nanomaterials-14-01019-s001.zip › nanomaterials-3044141-supplementary.pdf]

Supplementary Materials

Text S1: Modelling in COMSOL

The electric potential distribution of the triangular superlattice is numerically simulated in COMSOL. Figure S1 shows a unit cell of the triangular superlattice in the COMSOL model. Electric potential conditions are added at the top and middle of the model to simulate the gate voltage (V_g) and balance potential (V_b), respectively. The ground condition is applied at the bottom of the model to set a reference potential. Periodic boundary conditions are used around the model to simulate the periodic distribution of the superlattice potential. Parametric sweeps are conducted to vary the structural parameters of the superlattice, allowing for the investigation of superlattice potential distribution with different structural parameters.

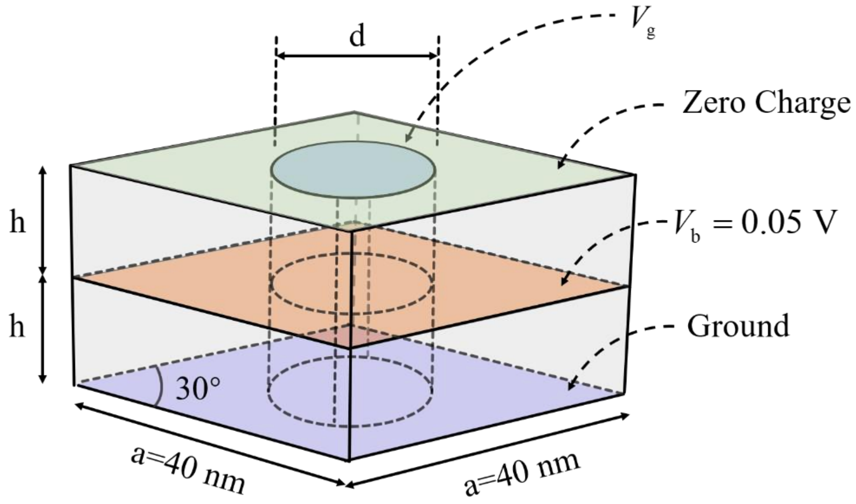

Figure S1. Schematic diagram of a unit cell of the triangular superlattice in COMSOL.

Text S2: Electrostatics Theory

For this model, the superlattice potential can be calculated based on Maxwell equations. The Maxwell equations that govern the electrostatic field in dielectric materials are satisfied by the following relation:

$$\nabla \times \vec{D} = \rho$$
$$\vec{E} = -\nabla V \quad \text{(S1)}$$

where $\vec{D}$ represents the electric displacement, ρ represents the charge density, $\vec{E}$ represents the electric field, V represents the electric potential.

The constitutive relationship for linear dielectric materials is:

$$\vec{D} = \varepsilon_0 \varepsilon_r \vec{E} \quad \text{(S2)}$$

where ε_0 represents the absolute permittivity, ε_r represents the relative permittivity.

At the material interface, we can derive the form of the boundary conditions from

Gauss's law as follows:

$$\vec{\mathbf{n}} \cdot \vec{D} = 0 \qquad \text{(S3)}$$

Text S3: Material properties

In the numerical simulations, the hBN layer is an idealized linear dielectric material with the relative permittivity of $\varepsilon_r = 5$.

In the COMSOL multiphysics settings, we set the balance potential V_{b} (the orange region in Figure S1) to 0.05 V, and the bottom of the hBN layer is grounded (the purple region in Figure S1). The period of the triangular superlattice a is 40 nm, and the diameter of the central circular region is d. The light green region in Figure S1 represents the zero charge condition, satisfying Equation (S3).

Through parametric sweeping, we can study the effects of the top gate voltage, duty cycle, and hBN thickness on the superlattice potential. The top gate voltage (V_{g}) is varied from -0.1 V to 0.2 V in steps of 0.05 V, the duty cycle (DC) is varied from 0.1 to 0.5 in steps of 0.2, and the hBN layer thickness (h shown in Figure S1) is varied from 5 nm to 15 nm in steps of 5 nm. In this paper, the duty cycle (DC) is defined as d / a.
